# Perioperative Management of Spontaneous Intracranial Hemorrhage in a Patient With Hemophilia A in a Resource Limited Country

**DOI:** 10.7759/cureus.43485

**Published:** 2023-08-14

**Authors:** Puneet Chopra, Manraj Singh, Adityabikram Singh, Athena Masi, Judith Yurkofsky, Brittany Zaita, Gurjinder Kaur

**Affiliations:** 1 Critical Care, Satguru Partap Singh (SPS) Hospital, Ludhiana, IND; 2 Basic Biomedical Sciences, Dayanand Medical College and Hospital, Ludhiana, IND; 3 Urology, Lenox Hill Hospital, New York City, USA; 4 Basic Biomedical Sciences, Touro College of Osteopathic Medicine, Middletown, USA

**Keywords:** india, resource limited, decompressive craniectomy, factor viii replacement, cryoprecipitate, perioperative management, spontaneous subdural hematoma, hemophilia a

## Abstract

Intracranial hemorrhage (ICH) is a serious complication of hemophilia A with high morbidity and mortality. The management of such cases is complicated by nonspecific and often delayed presentation, increased frequency of rebleeding, low awareness regarding clotting factor replacement, and debate regarding the efficacy of surgical interventions. We report a case of an 18-year-old male patient with hemophilia A, who first presented to the emergency department in India in a comatose state. Neuroimaging revealed subdural hematoma with midline shift and uncal herniation. The patient was successfully managed with perioperative cryoprecipitate and factor VIII replacement, tiered intracranial pressure lowering strategies, and early decompressive craniectomy with clot evacuation. In India, there are no standardized guidelines for screening and routine care for hereditary diseases like hemophilia. In a resource-deficient country, management was complicated by the limited availability of factor VIII in the emergent setting, as well as the inability to obtain serial factor levels in the postoperative period. We hope that this article helps to guide the management of ICH and hemophilia in resource-limited countries.

## Introduction

Hemophilia A is an X-linked recessive bleeding disorder estimated to be present in 1 in 5000 male live births [1]. Hemophilia A is diagnosed with the presence of low functional factor VIII activity in the presence of a normal von Willebrand Factor (vWF) level [2]. Initial screening test results for hemophilia A demonstrate normal platelet count, normal prothrombin time (PT) full form, and prolonged activated partial thromboplastin time (APTT) full form. Based on factor VIII level, Hemophilia A is classified into mild (6-30 IU/dl or 6-30% of normal), moderate (1-5 IU/dl or 1-5% of normal), or severe (less than 1 IU/dl or < 1% of normal) forms [3].

The most common signs and symptoms of hemophilia include spontaneous bleeds into muscles and joints, and prolonged bleeding times after minor traumas. In severe hemophilia cases, internal bleeding can be found in multiple organ systems and recurrent hemarthroses within the ankles, knees, and hips [2]. Intracranial hemorrhage (ICH) remains one of the worst events in such patients carrying significant morbidity and mortality risk in child and adolescent patients with hemophilia [2]. The incidence of ICH is 10-20-fold higher in hemophilia patients than in the general population and the risk factors are head trauma, thrombocytopenia, hypertension, previous ICH, or nonsteroidal anti-inflammatory drugs (NSAID) use [4]. The common sites of hemorrhage are subdural and intraparenchymal [4]. Due to non-specific initial symptoms of headache and vomiting, the diagnosis of ICH may often be delayed [4]. This was present in our case where our patient presented in a near comatose state four days after the initial onset of symptoms which were mistaken as a common migraine.

In the differential diagnosis of inherited bleeding disorders, one must also consider rarer causes such as hemophilia B and C. Hemophilia B presents in 1 in 30000 live male births and is diagnosed by identification of decreased factor IX clotting activity [3]. Hemophilia C is rare and usually presents as post-traumatic bleeding rather than spontaneous bleeding. It is diagnosed based on factor XI deficiency with typically prolonged APTT and normal PT on coagulation assay [5]. von Willebrand disease (vWD) and Factor V Leiden (FVL) are two other common congenital disorders that involve aberrations in coagulation cascade proteins. vWD is caused by deficient plasma von Willebrand factor (vWF), and unlike hemophilia patients who present with musculoskeletal bleeding, vWD patients primarily present with excessive mucocutaneous bleeding manifesting as epistaxis, gingival bleeds, and menorrhagia [6]. Factor V Leiden is an autosomal dominant clotting disorder that involves a mutation in the gene for factor V. Due to this factor V defect, patients are resistant to the action of activated protein C, increasing their risk of developing recurrent deep vein thrombosis (DVT) [7]. It is important to also keep other bleeding and clotting disorders on the differential in a case of an emergent intracranial bleed where the etiology may be unknown.

It is postulated that underdiagnosis, incomplete patient history, and mortality rates could explain lower hemophilia case reporting in India as compared to the United States because India lacks a national policy for the prevention and control of genetic disorders [8]. Adjusting for underdiagnosis of hemophilia A patients in India, the estimated number of patients in India is around 50,000 which means the country likely harbors the second-highest number of global patients with hemophilia A [8]. Therefore, this report aims to highlight the need for greater awareness of hemophilia, its complications, and management strategies specifically in a resource-limited country. The report also aims to emphasize the need for standardized long-term patient care to ensure disease optimization for this vulnerable population and decrease the incidence of fatal comorbidities such as ICH.

## Case presentation

**Figure 1:**
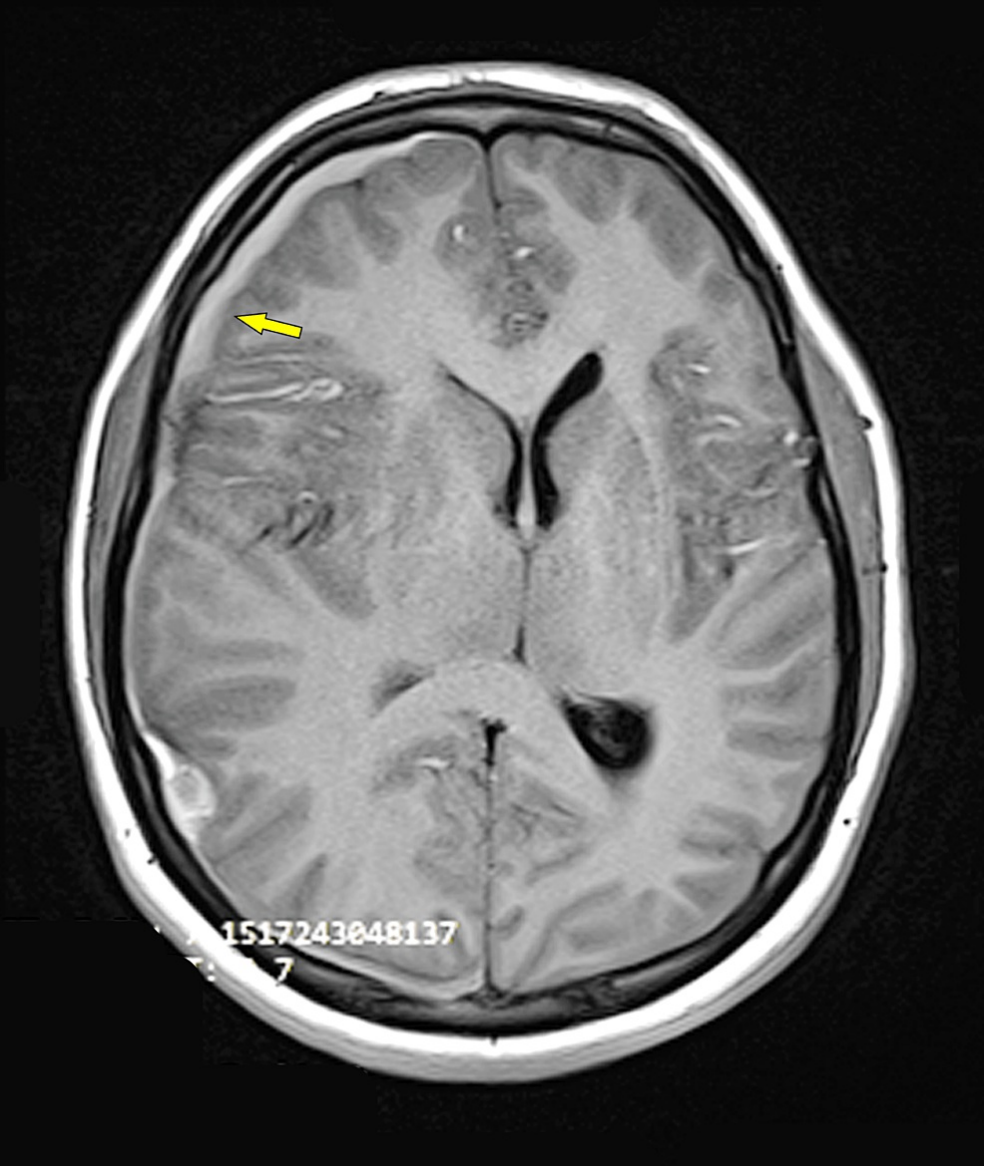
MRI axial T1 weighted image showing extra axial collection (subdural hemorrhage) along with right convexity (yellow arrow). Mass effect is seen compressing upon the right ventricle with a midline shift of 8.4 mm towards the left side.

**Figure 2:**
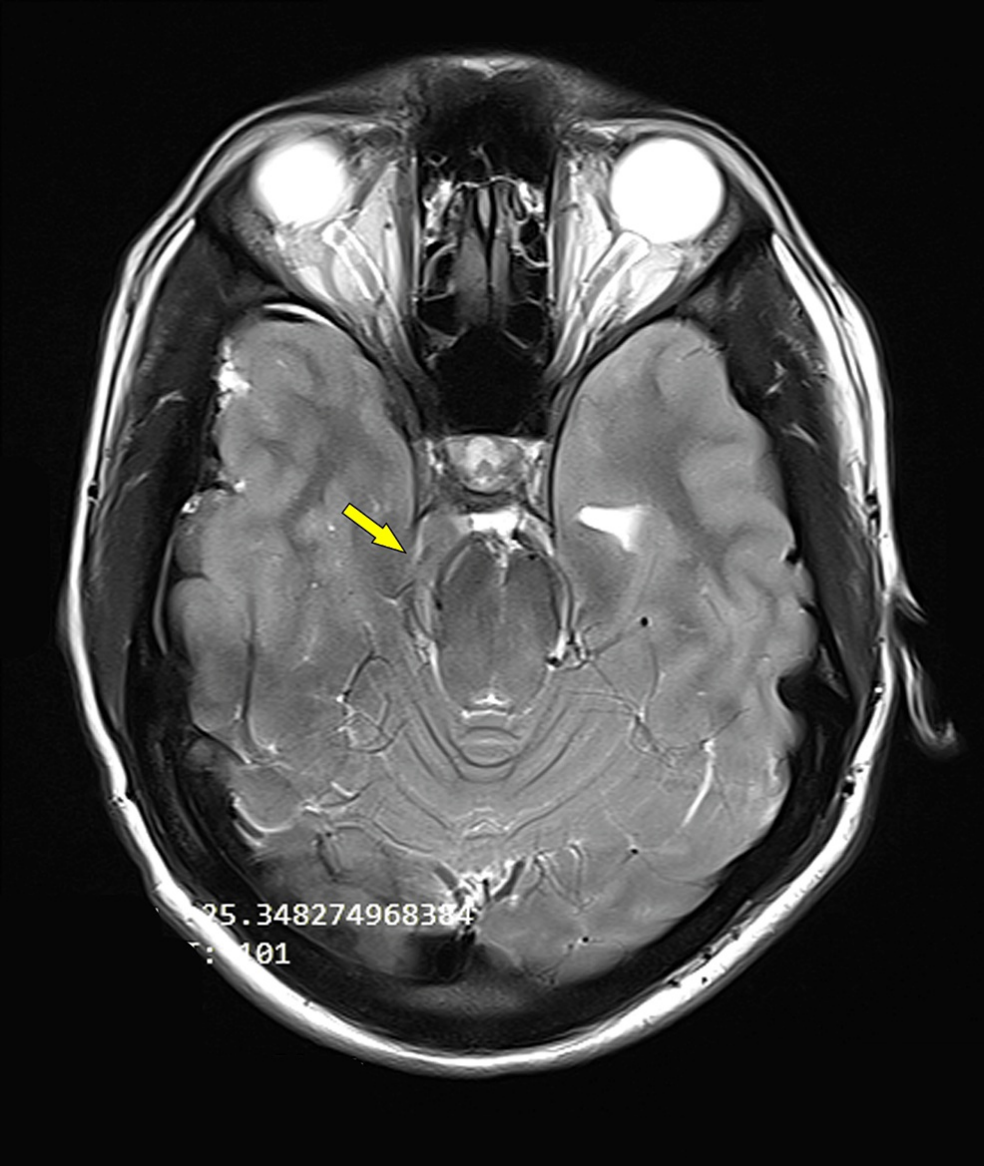
MRI axial T2 weighted image showing right uncal herniation (yellow arrow) compressing and displacing brainstem at the level of the midbrain towards the left side.

An 18-year-old male weighing 70 kg with a known history of hemophilia A, requiring recombinant factor VIII infusions biannually, presented to the hospital with complaints of headache, nausea, and recurrent vomiting for four days. One day prior to presentation at the hospital, the patient developed an altered sensorium. There was no recent history of head trauma or increased physical exertion, and no remote history of drug intake, previous surgery, or intracranial bleeding. On examination, the patient's Glasgow Coma Scale (GCS) was E1V1M2 and there was right-sided anisocoria. In view of low GCS, the patient was intubated with the use of intravenous (IV) fentanyl 100 mcg, propofol 80 mg, rocuronium 50 mg, and mechanically ventilated. The patient‘s hemoglobin was 12.4 g/dl, platelets were 4.94 lakh/cumm, prothrombin time (PT) was 15 seconds, and activated partial thromboplastin time (APTT) was prolonged at 100 seconds (Table 1). Magnetic resonance imaging (MRI) revealed acute to subacute subdural hematoma involving right cerebral convexity with mass effect causing compression over the ipsilateral lateral ventricle and midline shift of 8.2 mm towards the left side (Figure 1). MRI also revealed right uncal herniation with resultant compression of the midbrain region (Figure 2).

**Table 1:** Preoperative laboratory evaluation

Test	Result	Reference Range
Anti-HCV (rapid)	Non-Reactive	—
HbsAg (rapid)	Non-Reactive	—
HIV (rapid)	Non-Reactive	—
Aerobic C&S Blood, sample	Sterile after 5 days of aerobic incubation	—
Complete Blood Count		
Hemoglobin	12.4 g/dL (L)	13-17 g/dL
Total Leukocyte Count	21.6 x 10^3^/uL (H)	4-10 x 10^3^/uL
RBC Count	4.32 x 10^6^/uL (L)	4.5-5.5 x 10^6^/uL
ESR	35 mm/hr (H)	—
Packed Cell Volume	34.8% (L)	40-42%
Mean Corpuscular Volume	80.7 (L)	83-101 fl
Mean Corpuscular Hemoglobin	28.7	27-32 pg
MCHC	35.6 (H)	31.5-34.5 g/dL
RDW	14.7%	11-16%
Platelet	4.94 Lakhs/cumm (H)	1.5-4 Lakhs/cumm
MPV	11.1 fl	—
Neutrophils	86% (H)	40-80%
Lymphocytes	12% (L)	20-40%
Monocytes	1% (L)	2-10%
Eosinophils	1%	1-6%
PT/PT-INR		
PT	17.1 seconds (H)	11-15 seconds
Control	13.7 seconds	—
INR	1.25 seconds	—
aPTT/PTTK		
APTT	100 seconds (H)	25.4-38.4 seconds
Control	29.6 seconds	—
Liver Function Test/Profile-serum		
Total Bilirubin	1 mg/dL	0.2-1 mg/dL
Direct Bilirubin	0.1 mg/dL	0-0.2 mg/dL
Indirect Bilirubin	0.9 mg/dL	0-1 mg/dL
Total Protein	8.8 g/dL (H)	6.3-8.2 g/dL
Albumin	4.5 g/dL	3.5-5 g/dL
Globulin	4.3 g/dL (H)	2-3.5 g/dL
A/G Ratio serum	1.05 (L)	1.3-2
SGOT/AST	34	5-40 U/L
SGPT/ALT	37	5-40 U/L
Alkaline Phosphatase	113	46-116 U/L
GGT-serum	42	5-85 U/L
Renal Profile-serum		
Urea	38 mg/dL	15-39 mg/dL
Creatinine-serum	0.67 mg/dL	0.5-1.3 mg/dL
Uric Acid	4.7 mg/dL	2.5-7.5 mg/dL
Sodium (Na^+^) serum	132 mEq/dL (L)	135-145 mEq/L
Potassium (K^+^) serum	4.1 mEq/dL	3.5-5.1 mEq/L
Chloride (Cl^-^) serum	95 mEq/dL (L)	98-107 mEq/L
Calcium serum	9.6 mg/dL	8.5-10.1 mg/dL
Phosphorus serum	2.6 mg/dL	2.5-4.5 mg/dL

Intracranial pressure (ICP) lowering measures and clotting factor concentrate (CFC) replacement were initiated while the patient was prepared for emergency clot evacuation. Due to the non-availability of an adequate quantity of CFC pre-operatively, the patient was transfused 18 units of cryoprecipitate and 1750 units of intravenous recombinant factor VIII to correct coagulation abnormality. Following the improvement of the pre-operative APTT to 38 seconds, the patient underwent decompressive craniectomy with the insertion of an extra ventricular drain (EVD). The intra-operative course was uneventful with an estimated blood loss of 400 ml.

Post-operatively, the patient was transferred to the intensive care unit (ICU) for elective ventilation. The patient received recombinant factor VIII 3000 units intravenously q12h for the first three days, 2000 units q12h for the next four days, and 1500 units q12h for another seven days. Due to limited resources, we could measure factor VIII level of the patient only once (102% of activity on day two of the hospital stay) and regularly monitored APTT levels to assess coagulation status. Tranexamic acid was administered at the dosage of 500 mg IV q6h for seven days.

**Figure 3:**
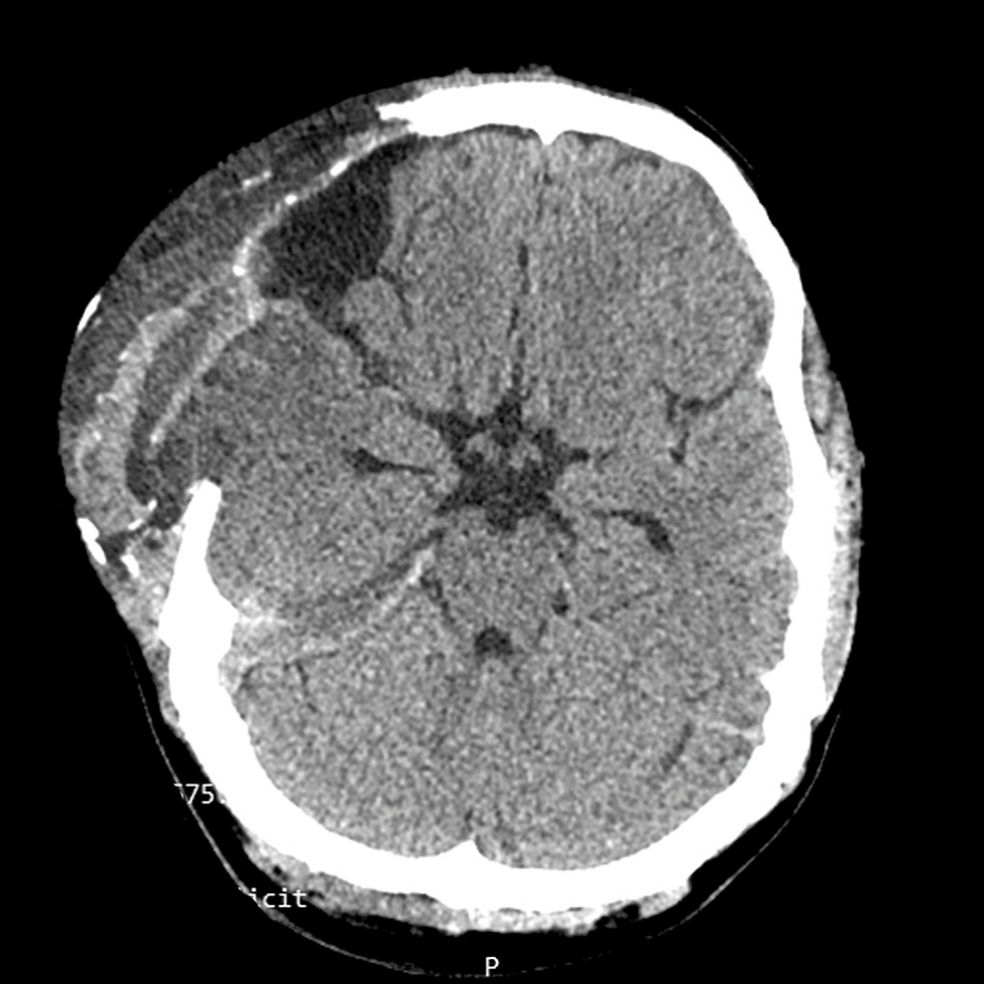
Post-operative CT showing residual clots with significant reduction in cerebral edema.

The patient was kept sedated in the early postoperative period. His ICP was monitored with EVD connected to a pressure transducer and episodes of raised ICP were managed with boluses of IV mannitol (0.5-1g/Kg) and temporarily opening EVD to drain cerebrospinal fluid. A postoperative CT scan revealed some residual clots with a significant reduction in cerebral edema (Figure 3). The patient was extubated on hospital day three in satisfactory condition with a GCS of 15/15 and improvement of the residual left-sided weakness over the course of his hospital stay.

Cranioplasty was performed uneventfully two months after the initial decompressive craniectomy procedure. The patient received perioperative factor VIII replacement during elective cranioplasty and made an excellent recovery.

## Discussion

It is known that high mortality is associated with spontaneous ICH, which leads to a dangerous clinical entity that requires prompt recognition and management [3,4]. This management is often complicated by variable presentation in patients with hemophilia as well as rapid deterioration that can occur secondary to herniation syndromes or mass effects from expanding hematomas [9]. To properly manage the hemophiliac patient with potential ICH, it is crucial to recognize and optimize risk factors for intracranial bleeding. One systematic review and meta-analysis studying 54,470 persons of all ages with hemophilia found that risk factors for ICH were advanced age, severe manifestations of the disease, HIV infection, presence of factor inhibitors, prior ICH, on-demand therapy, and African American ethnicity [10]. Additionally, hypertension remains the greatest risk factor for hemorrhagic events, especially in the aging population of hemophilia patients. Regular screening and proper management of comorbidities are the foremost components of ICH risk management in hemophilia patients.

The difficulty of our patient's presentation was that other than his known history of hemophilia, his symptoms leading up to the presentation were relatively benign. Additionally, this patient was relatively young and lacked the aforementioned risk factors which would predispose him to this event. Hemophiliac patients with ICH who present with headaches and minimal loss of consciousness have been successfully managed conservatively with blood products [11]. However, larger hemorrhages occurring in patients with multiple risk factors should always be monitored for signs and symptoms of mass effect or midline shift. In the emergent setting, pertinent signs and symptoms to review include Cushing’s triad, which describes the combination of bradycardia, irregular respirations, and hypertension in patients with elevated ICP [12]. Our patient showed features of increased ICP with definitive right uncal herniation on neuroimaging. The clinical manifestations of uncal herniation include a deterioration in consciousness, unilateral or bilateral dilatation of pupils, and hemiparesis. Brain herniation, if not managed quickly, leads to irreversible neurological deficits and high mortality [13]. Like cardiac arrest situations, acute brain herniations are “brain code” scenarios, which require immediate stepwise measures to reduce ICP including management of the airway, hyperosmolar therapy, control of agitation, and use of hyperventilation as the temporizing measures [12].

Additionally, early decompressive craniectomy, prior to the onset of significant neurological deficits, can be lifesaving in patients with uncal herniation by reversing secondary ischemia due to refractory intracranial hypertension [14]. Neurosurgical intervention was promptly considered in our young patient who was at high risk for devastating functional consequences and permanent neurologic deficits. Neurosurgery in hemophiliac patients is challenging due to the increased risk of perioperative bleeding. The various steps to prevent this complication include perioperative clotting factor replacement, maintenance of hemodynamics, and special care to achieve meticulous hemostasis during the intraoperative period [15]. It is important to prevent ICP elevations during the postoperative period, preferably guided by monitoring devices, as raised ICP is one of the important predictors of mortality [13].

It is critical to replenish factor VIII levels both pre and post-operatively to optimize the coagulation profile and reduce procedure-related complications including bleeding [16]. Another limitation of our study was that the patient was unable to have factor VIII level activity assessed due to resource limitations. Other examples from the literature show that without corrective infusions, factor VIII activity levels decrease precipitously following surgery [17]. This underscores the need to continue treatment with factor VIII in both the acute and subacute postoperative periods [17, 18]. In cases where factor levels are not available, an initial dose of 50 units/kg of factor VIII should be given for hemophilia A or 100-120 units/kg of factor IX for hemophilia B [19]. These doses assume negligible baseline factor levels. The inability to obtain factor levels greatly complicated post-operative monitoring for our patient. For this reason, ICP was very closely monitored with EVD, and any post-operative episodic elevations in ICP were promptly controlled with a hyperosmolar agent (mannitol).

Guidelines issued by the World Federation of Hemophilia specify the use of factor VIII concentrate, factor IX concentrate, cryoprecipitate, fresh frozen plasma (FFP), desmopressin, tranexamic acid, epsilon aminocaproic acid as the preferred products for the management of hemophilia and bleeding manifestations [16]. Factor replacement therapy with 100% correction within the first three days when supplemented with epsilon amino caproic acid, a potent antifibrinolytic, decreased mortality to only 10.8% in hemophilia patients with ICH [11]. Prior to the development of recombinant technology, cryoprecipitate was the primary blood product used to correct coagulation abnormalities in hemophilia patients [20]. Its use as a blood product has been declining since the 1990s because of safety concerns such as transmission of pathogens [20]. However, it is justified in emergent situations when there is a shortage of factor VIII CFC, as was in our case with our patient [18].

When concentrated clotting factors are not available or resource-limited, cryoprecipitate can be a useful product for controlling serious bleeding in the hemophiliac patient [18]. FFP is not recommended as cryoprecipitate is more concentrated than FFP and has a better ability to raise factor levels [18]. Furthermore, in patients with renal or heart disease, both cryoprecipitate and concentrated clotting factors are safer options than FFP due to decreased risk of volume overload [21]. Religious restrictions in various parts of the world can also impact the choice of blood products. Members of various religious groups can ethically reject transfusion products from other humans while others can refuse to be transfused with clotting factors made in animals such as pigs [22]. These are important points to consider when treating patients with hemophilia in a country as culturally and religiously diverse as India. 

Patients receiving these blood products frequently required them for extended courses of time. Our patient required treatment with recombinant factor VIII which was titrated down in dosage from 3000 units to 1500 units over two consecutive weeks. Similarly, 75% of patients from the Zanon et al*.* study required the use of blood products for at least three weeks. Furthermore, following their discharge from the hospital about 35% of patients were placed on lifelong prophylaxis with factor VIII infusions [23]. One proposed drawback of prophylactic factor infusions is the development of inhibitors; however, this was not observed in the study population [23]. Since the treatment of acute ICH in hemophilia patients requires optimization of the bleeding profile, prophylactic recombinant therapy for patients with severe disease or significant comorbidities such as hypertension may show clinical benefit. More robust data is needed to evaluate the benefit of prophylactic factor VIII infusions and their ability to avert serious bleeding complications such as spontaneous ICH in our patient population.

The healthcare system of the patient’s country of birth can frequently affect lifetime monitoring, management, and quality of life of patients with genetic conditions. In the United States, through national policies made by the Centers for Disease Control and Prevention (CDC), newborns are routinely screened for congenital disorders [24]. The United States population is ranked third largest in the world, compared to that of India’s population, which is ranked number one. Even with the increased population burden, India still does not have a national newborn screening (NBS) program as part of its health policy [25]. Fortunately, UNICEF reports that the percentage of Indian newborns who received routine postnatal care within two days of delivery has increased from 27.2% in 2016 to 81.6% in 2021 [26]. This statistic provides hope that vulnerable populations in developing countries can have access to similar levels of care in the management of congenital diseases. In the United States, through the use of NBS programs, testing for hemophilia may even be detected prenatally through cord blood sampling [27]. Through the study of genetic data obtained from standardized screening programs, the CDC reports about one-third of babies born with hemophilia have a novel mutation not present in other family members [28]. In a resource-limited country, advancements in the time to diagnosis and long-term management of these patients may, unfortunately, lag behind that of the United States, and this is an important consideration when evaluating the lifelong prognosis for our young patients.

An NBS program may prove to be highly beneficial in India for critical postnatal and preventative healthcare. Without proper documentation and available family history, this proposed NBS program may not meet its maximum potential. Lack of completeness, patient data mismatches, and the inability to share patient data across provincial healthcare systems plague the present health monitoring system [29]. This lack of proper documentation underlies India’s inefficiencies in detecting new cases of hemophilia and maintaining a working registry of disease. This lack of a central database may be responsible for multiple studies which show a mismatch between current data and the rising prevalence of hemophilia in the country. A study from 2014 by Kar et al*.* determined that over a five-year period, India and Brazil have reported more newly diagnosed patients than the USA or UK, reiterating the need for a prevention program [8]. A 2019 study with data from patients in Australia, Canada, France, Italy, New Zealand, and the United Kingdom determined that the prevalence of hemophilia at birth is higher than previously estimated; patients with hemophilia still have a life expectancy disadvantage. Establishing diagnoses at birth is a milestone toward assessing years of life lost, years of life with disability, and the total burden of disease [30].

## Conclusions

In conclusion, hemophilia A patients with ICH present with manifestations ranging from minimal loss of consciousness to a comatose state with brain herniation which requires multidisciplinary care by a neurologist, hematologist, neuro-intensivist, and neurosurgeon. We achieved excellent neurological recovery in our patient with the prompt institution of ICP lowering strategies, factor VIII replacement in the perioperative period, early clot evacuation with decompressive craniectomy in view of uncal herniation and ICP-guided management of intracranial hypertension postoperatively. However, the emergent management of this patient was complicated by resource limitations which led to the use of cryoprecipitate as adjunctive management, and careful ICP monitoring in the absence of post-operative factor levels. In the future, the authors hope that a rise in the prevalence of hemophilia in India will prompt greater recognition and management with formal governmental policy.
